# First-line pembrolizumab plus androgen deprivation therapy for locally advanced microsatellite instability-high prostate cancer in a patient with Muir-Torre syndrome: A case report

**DOI:** 10.3389/fonc.2023.1126476

**Published:** 2023-03-03

**Authors:** Mohammad O. Atiq, Danielle M. Pastor, Fatima Karzai, Amy R. Hankin, Baris Turkbey, Lisa M. Cordes, Isaac Brownell, Yi Liu, Gregory T. Chesnut, Ravi A. Madan

**Affiliations:** ^1^ Genitourinary Malignancies Branch, Center for Cancer Research, National Cancer Institute, National Institutes of Health, Bethesda, MD, United States; ^2^ Center for Immuno-Oncology, Center for Cancer Research, National Cancer Institute, National Institutes of Health, Bethesda, MD, United States; ^3^ Molecular Imaging Branch, Center for Cancer Research, National Cancer Institute, National Institutes of Health, Bethesda, MD, United States; ^4^ Dermatology Branch, National Institute of Arthritis and Musculoskeletal and Skin Diseases, National Institutes of Health, Bethesda, MD, United States; ^5^ Genetics Branch, Center for Cancer Research, National Cancer Institute, National Institutes of Health, Bethesda, MD, United States; ^6^ Urology Service, Department of Surgery, Walter Reed National Military Medical Center, Bethesda, MD, United States; ^7^ Center for Prostate Disease Research, Murtha Cancer Center Research Program, Department of Surgery, Uniformed Services University of the Health Sciences, Bethesda, MD, United States

**Keywords:** Muir-Torre Syndrome, lynch syndrome, prostate cancer, immunotherapy, microsatellite instability (MSI), mismatch repair genes, immune checkpoint inhibitor, androgen deprivation therapy (ADT)

## Abstract

The risks of development of colorectal and endometrial cancers in individuals with Lynch syndrome (LS) are well known and have been widely studied. In recent years, the potential association of other malignancies, including prostate cancer, with LS has been considered. Decision-making regarding screening for prostate cancer in the generalized population can be complicated; accounting for the possibility of a higher risk of cancer conferred by a potential genetic predisposition confounds the creation of salient guidelines even further. Although tissue-agnostic treatment approvals have been granted to several immune checkpoint inhibitors (ICIs) for their use in the treatment of subsets of patients whose tumors exhibit high levels of microsatellite instability or high tumor mutational burden, a paucity of data exists regarding the use of ICIs in the first line treatment of patients with locally advanced prostate cancer harboring these features. A significant reduction in tumor volume in response to the combination of immune checkpoint inhibition and androgen deprivation therapy is described in this report of a male with Muir-Torre syndrome who was found to have locally advanced adenocarcinoma of the prostate. While anecdotal, the anti-tumor activity of this combination of therapy is notable and calls attention to the importance of considering further investigation of the use of immune checkpoint blockade as a primary therapeutic option in patients with localized prostate cancer.

## Introduction

Pembrolizumab is a programmed cell death-1 (PD-1) receptor blocking antibody that is authorized for use by the U.S. Food and Drug Administration for a number of indications in the treatment of solid tumor malignancies, including use as monotherapy, in combination with chemotherapeutic agents, and in conjunction with targeted therapy (1). Its role as a therapeutic option for individuals with prostate cancer is limited, however, and extends only to the small subset of patients whose tumors exhibit high levels of microsatellite instability (MSI-H), are deficient in mismatch repair (dMMR), and/or express high tumor mutational burden (TMB-H). Lynch syndrome (LS) refers to an autosomal dominant disorder associated with inactivating germline mutations in DNA MMR genes or structural variants at the *EPCAM* locus silencing MSH2 protein expression. Individuals with pathogenic germline variants of these MMR gene mutations are predisposed to the development of multiple types of cancers throughout their lifetime (2). Malignancies that develop in this setting typically exhibit high levels of MSI, secondary to impaired gene replication and disrupted DNA homeostasis which lead to genetic hypermutability and changes in microsatellite length (3). In recent years, prostate cancer has increasingly been suggested as a tumor type associated with this syndrome (4–7). Although literature exists regarding the use of pembrolizumab in patients with LS and metastatic prostate cancer, to our knowledge, there have not been any reports to date describing the use of pembrolizumab in the first-line setting for LS patients with locally advanced prostate cancer. In this report, we describe a patient with locally advanced, MSI-H prostate cancer in the setting of Muir-Torre syndrome (MTS), a rare phenotypic variant of LS associated with sebaceous carcinoma risk, who has experienced a robust response to first-line treatment with the combination of pembrolizumab and androgen deprivation therapy (ADT). Although dMMR/MSI-H cancers of the prostate are, overall, uncommon, the impact of this molecular phenotype on clinical decision-making for those patients with tumors harboring these features is significant.

## Case description

A 70-year-old man developed lower urinary tract symptoms. His past medical history was notable for a low-grade urothelial carcinoma of the bladder (treated with transurethral resection of bladder tumor and intravesical BCG), right-sided colon cancer (treated with hemicolectomy and systemic 5-fluorouracil), squamous cell carcinoma of the tonsil (treated with resection and systemic cisplatin, followed by chemoradiation with 5-fluorouracil as a radiosensitizing agent), and numerous cutaneous squamous cell carcinomas and sebaceous carcinomas. A strong family history of cancer was evident and included a daughter with metastatic ovarian cancer in the setting of MTS, a father with brain and duodenal cancers, and a half-sister with MTS. Family history was also notable for multiple siblings with various malignancies, including cervical, colon, and oral cancer of unknown type.

**Figure 1:**
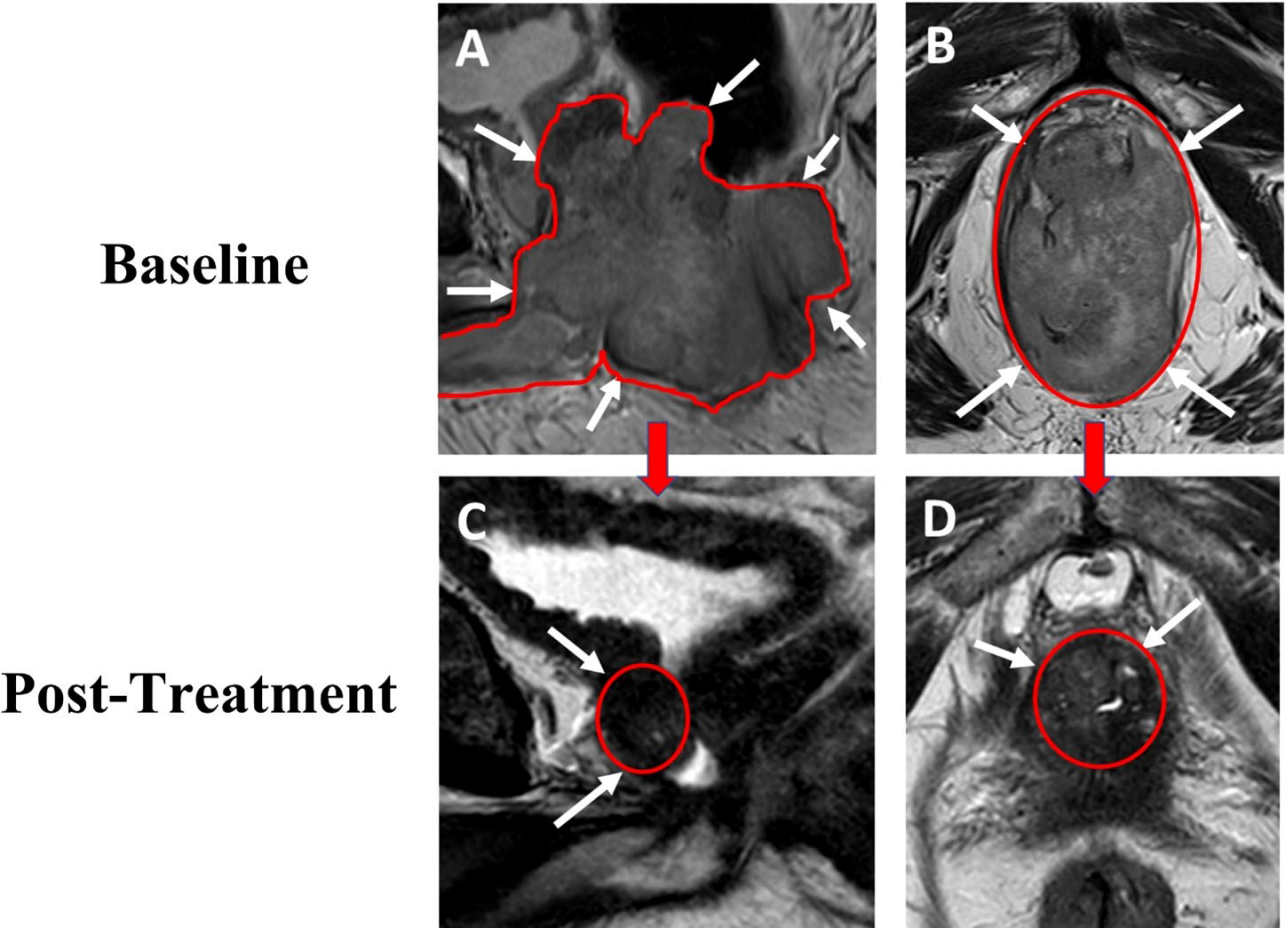
69-year-old male with advanced prostate cancer. Baseline sagittal **(A)** and axial **(B)** T2W MRI performed after transurethral resection show a large mass (total lesion volume of 282cc) occupying the whole prostate with rectum, bladder with diffusion restriction on ADC map. Post-treatment follow up sagittal **(C)** and axial **(D)** T2W MRI show a gradual decrease in size of the lesion (total lesion volume 11.4cc) without evidence of rectum and bladder involvement. T2W, T2 weighted image; ADC, apparent diffusion coefficient.

Shortly after the onset of his urinary symptoms, the patient developed intermittent bowel incontinence and gross hematuria. He presented for evaluation and a transurethral resection of the prostate was recommended. He ultimately underwent transurethral resections of the prostate, bladder neck, and prostatic urethra, all specimens of which were confirmed to be prostatic adenocarcinoma with features consistent with very high risk prostate cancer (Gleason 5 + 4, with 95% grade group 5, with >80% involvement of tumor within resected tissue and extraprostatic extension [EPE] into the lamina propria of the urothelium). Positive expression of both prostate-specific antigen (PSA) and NKX3.1 in all specimens was determined by immunohistochemical (IHC) evaluation. Serum PSA level was found to be 12.36 ng/mL. Magnetic resonance imaging (MRI) of the pelvis that was performed after transurethral resection revealed circumferential involvement of the rectum with extension into the base of the penis with no lymphadenopathy (**Figure 1**). No visceral nor bone metastases were demonstrated on computed tomography scan nor bone scintigraphy, respectively. The patient then underwent a colonoscopy with biopsy of rectal mass, as well as repeat biopsies of the bladder trigone and prostatic urethra; all specimens exhibited positive expression of PSA, NKX3.1, and CDX2 by IHC evaluation, further supporting the diagnosis of invasive prostatic adenocarcinoma. Additional analysis of the original prostate pathology specimen revealed loss of nuclear positivity of MSH2 and MSH6 with intact nuclear positivity of MLH1 and PMS2. Microsatellite testing by PCR revealed the specimen to be MSI-H. Prior germline genetic testing had established that the patient carried a pathogenic variant in c.319delinsAATAAGGCATC (p.Ala107fs) in the *MSH2* gene and a variant of uncertain significance c.1816C>T (p.Pro606Ser) in the *BRCA2* gene.

**Figure 2:**
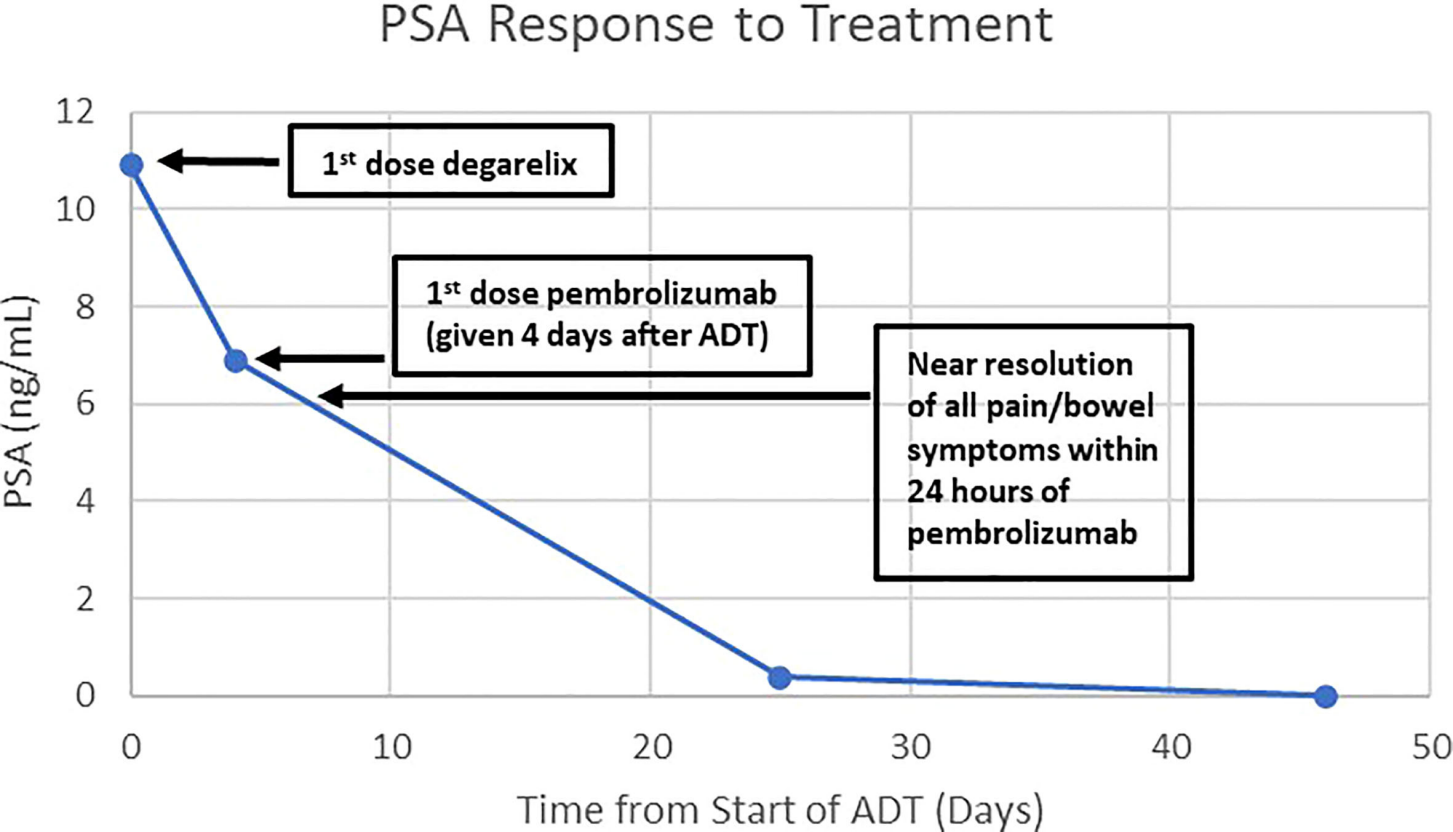
Graph of the patient’s PSA response to treatment with degarelix and pembrolizumab. The patient was treated with degarelix (denoted at Day 0) when his PSA was 10.92 ng/mL. Four days later, he was given pembrolizumab when his PSA was 6.9 ng/mL. His symptoms from the prostate mass largely resolved within 24 hours of the first dose of pembrolizumab. The PSA became undetectable at day 46 from start of degarelix.

One month following his transurethral resections, the patient developed new symptoms of coccydynia, constipation, bowel incontinence, and change in stool caliber. Serum PSA level at this time was 10.92 ng/mL. The extent of local disease and his other comorbidities, including congestive heart failure, atrial fibrillation, and asthma, prompted physicians to seek potential alternative initial treatment strategies to radiation treatment or operative intervention, particularly given the molecular characteristic of MSI-H disease. He was evaluated at the National Cancer Institute of the National Institutes of Health and, in consideration of the dMMR and MSI-H status of his tumor, treatment was initiated with pembrolizumab, in combination with ADT without concomitant androgen blockers. Treatment was comprised of pembrolizumab 200 mg administered intravenously every 3 weeks, with degarelix 240 mg loading dose given as 2 subcutaneous injections followed by a singular subcutaneous 80 mg maintenance dose given every 4 weeks, prior to eventually transitioning to leuprolide acetate for depot suspension 22.5 mg given as a single intramuscular injection every 12 weeks. Within 24 hours of initial pembrolizumab infusion, the patient reported near-complete resolution of straining with defecation, with marked improvement of urinary symptoms. Within one month of initiation of therapy, his serum PSA level was undetectable (**Figure 2**). Multiparametric MRI of the prostate was obtained after 2 cycles of treatment with pembrolizumab plus ADT and showed a decrease in size in the intraprostatic lesion, from 8.9 cm to 2.8 cm (**Figure 1**). This radiographic finding corresponded to a reduction in prostate volume from an original total lesion volume of 282 cc to a post-treatment volume of 11.4 cc. Extraprostatic extension was evident posteriorly and laterally, to the right of the prostate; invasion of the seminal vesicles was visible at the root. Notably, the tumor involvement of the bladder and rectum that had been previously identified was no longer visualized. The patient was continued on pembrolizumab plus ADT. A subsequent MRI of the prostate was performed at six months of therapy and demonstrated a further decrease in the greatest dimension of the intraprostatic lesion at 2.1 cm. Additionally, EPE had resolved per imaging.

During his treatment, the patient developed verrucous cutaneous lesions. Dermatology was consulted and a biopsy was performed which confirmed benign lichenoid keratoses, a known dermatologic effect of pembrolizumab. The patient’s PSA level has remained undetectable at 19.5 months since initiation of treatment and continuation of pembrolizumab plus ADT is planned for a total of 2 years, with serial monitoring of his PSA level and repeated MRI of the prostate to be performed at scheduled intervals.

## Discussion

Lynch syndrome is an inherited disorder known to confer an increased lifetime risk of the development of several types of cancers to those individuals harboring pathognomonic germline mutations of genes associated with dysfunctional DNA MMR. Although the associations of colorectal and endometrial malignancies with this syndrome have long been well-established, estimated risks of other tumor types have also been demonstrated to be higher than those posed to the general, unaffected population. Gastric, intestinal, hepatobiliary, pancreatic, and epithelial ovarian cancers have been accepted as constituent malignancies of the syndrome, as have certain genitourinary malignancies, of which renal pelvic, ureteral, and bladder have been recognized. In recent years, however, prostate cancer has also been proposed to be linked with LS (7, 8).

The increased risk of cancer development in LS is attributed to the presence of germline mutations in DNA MMR genes (*MLH1, MSH2, MSH6, PMS2, EPCAM)*), which are propagated through an autosomal dominant pattern of inheritance (9). The majority of resultant tumors exhibit MSI, a tumor characteristic now recognized to be associated with immunotherapy responsivity (10, 11). Similarly, tumors exhibiting MSI caused by sporadic, acquired hypermethylation of the promoter of the *MLH1* gene also seem to demonstrate improved responses to immune-based therapies (12, 13).

The recognition of the relevance of somatic mutations of DNA repair pathway genes in individuals with prostate cancer to treatment response has not only impacted therapy algorithms but has also influenced recommendations and guidelines regarding germline testing in this patient population (14–17). Multiple studies have demonstrated that proportions of individuals with prostate tumors expressing somatic mutations in either homologous recombination DNA repair pathway genes or genes governing mismatch base excision repair also carry related germline mutations (18–21). These discoveries have led to increasing efforts to identify the risk of prostate cancer development in individuals with LS. Through the evaluation of more than 15,000 various types of tumors, Latham et al. have suggested that MSI/dMMR may be predictive of LS across a more extensive range of cancer type than traditionally appreciated, with 5% of 1048 patients with prostate cancer determined to exhibit MSI-H or MSI-indeterminate (MSI-I) tumors, of which 5.6% were found to have LS (10). In a similar effort to determine the prevalence of MSI in prostate cancer, other investigators found that 3.1% of examined prostate tumors exhibited MSI-H or dMMR; of the patients diagnosed with these tumors, 21.9% carried a pathogenic germline mutation in a LS-associated gene, with mutations in *MSH2* most frequently expressed (4).

In an international prospective, targeted prostate cancer screening study in men aged 40-69 years of age considered to be at genetically higher risk of developing prostate cancer than age-matched controls, more than 600 of 828 males from LS families were found to have germline pathogenic variants in *MLH1*, *MSH2*, and *MSH6* genes (5). These individuals were to undergo annual PSA screening for a minimum of 5 years, with transrectal, ultrasound-guided prostate biopsy recommended for PSA concentration of higher than 3.0 ng/mL. Researchers diagnosed prostate cancer in 4.3% of 305 men with *MSH2* mutations and 3% of 135 men with *MSH6* mutations; conversely, only one of 210 (0.5%) non-carriers in the *MSH2* control group and none of the 177 non-carriers in the *MSH6* control group were found to have prostate cancer. Individuals with *MSH2* mutations were eight times more likely to be diagnosed than their non-carrier counterpart and were diagnosed at younger ages (an average of 58 years versus 66 years, respectively). Further, males with *MSH2* mutations diagnosed with prostate cancer were found to have more aggressive disease than matched control. Patients harboring *MSH6* mutations with prostate cancer were diagnosed at an average age of 62 years; 75% of these individuals were determined to have aggressive disease. Of note, 9 individuals with *MSH2* gene mutations found to have PSA levels greater than 3 ng/mL did not proceed to biopsy, while 5 were found to have benign tissue on biopsy; of the *MSH2* non-carrier controls, 4 who met PSA criteria did not undergo biopsy, while benign tissue was found on biopsies of 2 individuals (5).

The findings in the above studies suggest increased risk for the development of prostate cancer in individuals with LS and underscore the importance of early screening in this population particularly as no consensus currently exists regarding screening in this setting. Currently, prostate cancer screening is not recommended for the general population; rather, the National Comprehensive Cancer Network (NCCN) guidelines suggest that the decision to test baseline PSA level follows an informative discussion between physicians and healthy patients between the ages of 40 - 75 years who are at higher than average risk, such as those with strong family history of prostate cancer, who carry germline mutations that may increase the risk of prostate cancer, and/or with African ancestry (22).

The clinical course of the patient described in this report was further complicated by the presence of MTS. Rarely, LS may manifest with or involve a dermatologic phenotype, with the development of sebaceous adenomas, epitheliomas or carcinomas, and/or keratoacanthomas. Muir-Torre syndrome is a rare variant of LS, more commonly diagnosed in men, with individuals characteristically developing at least one cutaneous tumor and at least one visceral neoplasm (most commonly of gastrointestinal origin, with genitourinary malignancies occurring with second most common frequency) (23). Germline mutations in *MLH1*, *MSH2*, and *MSH6* genes have been implicated in MTS, with *MSH2* mutation occurring most frequently, and both *MLH1* and *MSH2* mutations being associated with more aggressive phenotypes (24, 25).

Treatment of prostate cancer with immune checkpoint blocking monotherapy has been largely unsuccessful, except in a small subset of patients. Two immunotherapies, pembrolizumab and sipuleucel-T, carry indications for their use in the metastatic setting. However, these indications are specific to metastatic castrate-resistant prostate cancer (mCRPC) patients whose tumors are MSI-H/dMMR/TMB-H or to those who have no visceral involvement, respectively. Anti-tumor activity attributed to pembrolizumab has been described in patients with LS with metastatic MSI-H/dMMR prostatic cancer (4, 26). However, as previously mentioned, no reports of checkpoint inhibition as first-line treatment of locally advanced prostate-cancer have been described to our knowledge.

The reduction in the patient’s tumor volume resulting from the combination of treatment with pembrolizumab and ADT is more robust than the response expected from treatment with ADT alone. A study of neoadjuvant hormonal therapy given prior to radiation showed that patients administered goserelin for an average of 192 days had a mean prostate volume reduction of 26% (27). The addition of androgen receptor inhibition has been shown to reduce prostate volume to a similar degree. A randomized study examining the resultant prostate volume of patients with localized prostate cancer treated with an average of 3 months of neoadjuvant bicalutamide monotherapy versus bicalutamide plus ADT prior to radiation showed that the mean volume reduction in the monotherapy arm was 17.5%, compared to 28% for the combined therapy arm (28). In a comparison of degarelix versus goserelin, with each administered over 3 months in the neoadjuvant setting, the mean percentage reduction in prostate volume was demonstrated to be -36.0% ± 14.5% versus -35.3% ± 16.7%, respectively (29). These results suggest that an appropriate anticipated estimate for ADT-induced tumor volume reduction might be between 20-40% over 3 months (or, in less stringent terms, a maximal expectation of 50% reduction in tumor volume). Our patient’s reduction in tumor volume at 2 months from initiation of ADT was 95.96%. This magnitude of volume reduction is substantially greater than that expected from ADT alone, suggesting that the afforded benefit may essentially be derived from the addition of anti-PD-1 therapy. This also raises the possibility to revisit radiation therapy now that his locally advanced disease appears more localized.

It is uncertain if ADT was essential for the extent of response demonstrated in the individual described herein. Existing data support that ADT can enhance CD8^+^ T cell infiltration in the prostate tumor microenvironment (30–34). However, a descriptive study conducted by Sommer et al. could not confirm increases in PD-L1 expression after ADT (34). Furthermore, a phase 3 study of patients who would have recently started an ADT-based regimen for newly diagnosed metastatic castration sensitive disease failed to demonstrate that pembrolizumab could improve clinical outcomes over ADT-based therapy, adding to the list of failed PD-1/PD-L1 inhibition trials in prostate cancer (35). Further investigations are required to determine the role that ADT may play in enhancing immunotherapy efficacy in patients with certain genetic mutations such as this patient and if outcomes would be different than in unselected populations.

Recently published data from clinical trials evaluating neoadjuvant immune checkpoint blockade in the treatment of non-metastatic dMMR colon and rectal cancers have illuminated the impact of the “immunoablative” effect of these agents on the potential obviation of additional modalities such as chemoradiotherapy and surgery (36, 37). A prospective phase II study in which single agent dostarlimab, an anti-PD-1 monoclonal antibody, was administered to patients with dMMR locally advanced rectal cancer for six months as neoadjuvant therapy has resulted in striking findings (36). Investigators observed that all 12 patients who had completed treatment and had undergone at least 6 months of follow-up demonstrated clinical complete response, with no evidence of tumor on magnetic resonance imaging, ^18^F-fluorodeoxyglucose–positron-emission tomography, endoscopic evaluation, digital rectal examination, or biopsy. Patients who achieved clinical complete response were eligible for omission of chemoradiation and surgery; at the time of publication of results, no patients had required chemoradiotherapy or surgery, nor had any cases of disease progression or recurrence been reported during a follow-up period ranging from 6 - 25 months. In the NICHE-2 study, patients with non-metastatic dMMR colon cancer were treated with one dose of ipilimumab and two doses of nivolumab and underwent surgery ≤ 6 weeks of registration (37). With a median time from first dose to surgery of 5 weeks, pathologic response (defined as ≤50% residual viable tumor) was observed in 106/107 (99%) patients, with 102/107 (95%) exhibiting a major pathologic response (defined as ≤10% residual viable tumor), including 67% demonstrating a complete pathologic response. At a median follow-up of 13 months, none of the patients had developed recurrent disease. While longer follow-up is warranted to assess duration of response in these studies, these findings support the movement towards a potential paradigm shift through which immunotherapy could be used at earlier stages of disease in effort to maximize organ-sparing approaches as treatment strategies. Similarly, the possibility that treatment of dMMR/MSI-H locally advanced prostate cancer with immunotherapy could eliminate the need for radical surgery is intriguing, given the emerging data described regarding the use of ICIs in locally advanced dMMR CRC.

## Conclusion

The role of checkpoint inhibition in the treatment of prostate cancer remains limited to a select subpopulation of patients. While currently approved indications and published studies relate to mCRPC, there exists potential for utilizing immunotherapeutic agents earlier in the disease process for patients with genetic aberrations such as those seen in LS. By utilizing pembrolizumab in the neoadjuvant setting, physicians may be able to exploit the aforementioned underlying tumor biology in LS patients to yield an augmented reduction in tumor burden as compared to that associated with ADT monotherapy. Further, it is tantalizing to understand how much tumor the patient described still has and/or how long his disease control could continue from the treatment of ADT and pembrolizumab. It also remains unclear if ADT was required for this response or if the patient needs to remain on ADT indefinitely and what his duration of therapy with ICI should be. Subgroups of patients such as those with dMMR/MSI-H locally advanced disease who may not be considered candidates for local treatment and whose tumors exhibit particular molecular features may experience greater benefit from immune checkpoint inhibition than that expected with ADT alone. Future studies in populations with dMMR/MSI-H, such as individuals with LS, should be considered to evaluate the clinical benefit of immune checkpoint inhibition in the setting of locally advanced prostate cancer.

## Data availability statement

The original contributions presented in the study are included in the article/supplementary material, further inquiries can be directed to the corresponding author/s.

## Ethics statement

Written informed consent was obtained from the individual(s) for the publication of any potentially identifiable images or data included in this article.

## Author contributions

MA and DP contributed equally to this work and share first authorship. Conception/Design: MA, DP, FK, RM; Provision of study material or patients: MA, DP, FK, AH, BT, LC, IB, YL, GC, RM; Collection and/or assembly of data: MA, DP, AH, BT; GC; Data analysis and interpretation: BT, IB, YL, FK, RM; Manuscript writing and/or editing: MA, DP, FK, BT, LC, IB, RM; Final approval of manuscript: MA, DP, FK, RM. All authors were responsible for the contribution of important intellectual content. All authors contributed to the article and approved the submitted version.
